# Associations of HLA alleles with specific language impairment

**DOI:** 10.1186/1866-1955-6-1

**Published:** 2014-01-17

**Authors:** Ron Nudel, Nuala H Simpson, Gillian Baird, Anne O’Hare, Gina Conti-Ramsden, Patrick F Bolton, Elizabeth R Hennessy, Anthony P Monaco, Julian C Knight, Bruce Winney, Simon E Fisher, Dianne F Newbury

**Affiliations:** 1 Wellcome Trust Centre for Human Genetics, University of Oxford, Oxford OX3 7BN, UK; 2 Newcomen Centre, the Evelina Children’s Hospital, Westminster Bridge Road, London SE1 7EH, UK; 3 Department of Reproductive and Developmental Sciences, University of Edinburgh, 20 Sylvan Place, Edinburgh EH9 1UW, UK; 4 School of Psychological Sciences, University of Manchester, Oxford Road, Manchester M13 9PL, UK; 5 Departments of Child & Adolescent Psychiatry & Social Genetic & Developmental Psychiatry Centre, Institute of Psychiatry, King’s College London, De Crespigny Park, London SE5 8AF, UK; 6 University Child Health and DMDE, University of Aberdeen, Foresterhill, Aberdeen AB25 2ZG, UK; 7 Tufts University, Ballou Hall, Medford, MA 02155, USA; 8 Department of Oncology, University of Oxford, ORCRB, Roosevelt Drive, Oxford OX3 7DQ, UK; 9 Max Planck Institute for Psycholinguistics, Wundtlaan 1, 6525 XD, Nijmegen The Netherlands; 10 Donders Institute for Brain, Cognition and Behaviour, Radboud University, Geert Grooteplein-Noord, 6525 EZ, Nijmegen, The Netherlands; 11 A list of SLIC members can be found in the acknowledgements section

**Keywords:** Specific language impairment (SLI), HLA, Neurodevelopmental disorders, Genetic association

## Abstract

**Background:**

Human leukocyte antigen (HLA) loci have been implicated in several neurodevelopmental disorders in which language is affected. However, to date, no studies have investigated the possible involvement of HLA loci in specific language impairment (SLI), a disorder that is defined primarily upon unexpected language impairment. We report association analyses of single-nucleotide polymorphisms (SNPs) and HLA types in a cohort of individuals affected by language impairment.

**Methods:**

We perform quantitative association analyses of three linguistic measures and case-control association analyses using both SNP data and imputed HLA types.

**Results:**

Quantitative association analyses of imputed HLA types suggested a role for the *HLA-A* locus in susceptibility to SLI. *HLA-A* A1 was associated with a measure of short-term memory (*P* = 0.004) and A3 with expressive language ability (*P* = 0.006). Parent-of-origin effects were found between *HLA-B* B8 and HLA-DQA1*0501 and receptive language. These alleles have a negative correlation with receptive language ability when inherited from the mother (*P* = 0.021, *P* = 0.034, respectively) but are positively correlated with the same trait when paternally inherited (*P* = 0.013, *P* = 0.029, respectively). Finally, case control analyses using imputed HLA types indicated that the DR10 allele of *HLA-DRB1* was more frequent in individuals with SLI than population controls (*P* = 0.004, relative risk = 2.575), as has been reported for individuals with attention deficit hyperactivity disorder (ADHD).

**Conclusion:**

These preliminary data provide an intriguing link to those described by previous studies of other neurodevelopmental disorders and suggest a possible role for HLA loci in language disorders.

## Background

The human major histocompatibility complex on chromosome 6 contains many highly polymorphic genes involved in immune function [1,2], notably the human leukocyte antigen (HLA) genes. Specific HLA alleles have been associated with more than 100 traits, including striking association with autoimmune diseases [3,4]. Given the identification of possible abnormal immune responses observed in participants with autism (for example, antibodies which react to proteins found in the central nervous system [5]) and similarities between schizophrenia and diseases with established HLA associations [6], several studies have examined the possible involvement of HLA loci in neurodevelopmental disorders. Such investigations have typically targeted autism and attention deficit hyperactivity disorder (ADHD) as well as psychiatric disorders such as schizophrenia. Specific alleles (please see a note regarding the terminology in the Methods section) of *HLA-DRB1*, including DR4 and DR12, have been associated with autism, ADHD, and schizophrenia, although these findings have not always replicated within or between disorders. In addition, association between the A2 allele of the *HLA-A* gene and autism has been reported [7].

Pertinent to the present study is the suggestion that autoimmunity may also be involved in neurodevelopmental disorders that include language deficits: a positive autoimmune reaction to myelin was detected in children with Landau-Kleffner syndrome, a disorder characterized by aphasia and epileptic seizures [8]. Autoantibodies that react with brain tissue have been found in a relatively large number of children with an atypical variant of Landau-Kleffner syndrome and autism compared with controls. Such antibodies were also found in children affected by other neurological disorders, albeit less frequently [9]. Another study found antibodies that bound to rodent Purkinje cells in the serum of a mother with a child with specific language disorder, a child with autism, and a child with typical development. When this serum was injected in pregnant mice, the mouse pups had reduced exploratory behavior and impaired motor coordination [10]. The HLA region has also been implicated in a linkage study of dyslexia [11].

Specific language impairment (SLI) is diagnosed when a child has problems with the acquisition of language but shows normal development in all other areas [12]. SLI is a heterogeneous and complex disorder with a strong genetic component [13]. Several linkage regions and genes have been identified in genetic studies of SLI [14-17]. One gene in particular, *CNTNAP2* (contactin associated protein-like 2), has been implicated in SLI, autism, and ADHD [16,18,19]; in the context of SLI, this gene was associated across three language-related measures: nonword repetition (NWR) scores, Clinical Evaluation of Language Fundamentals (CELF) expressive language scores (ELS), and CELF receptive language scores (RLS) [16], all of which have been employed in the present study. The product of the *CNTNAP2* gene is involved in forming the gaps in the myelin sheath [20], which is interesting in the context of the autoimmune reaction to myelin detected in children with Landau-Kleffner syndrome. Though clinically distinct, SLI, autism, and ADHD have some phenotypic similarities; language may be impaired in all three disorders [21-23]. The core linguistic deficit in SLI may not be the same as the one in autism or ADHD. Children with ADHD and children with SLI may differ in their conversational profiles. Whereas children with SLI tend to have more limitations in the areas of lexical diversity, sentence length, and morphosyntactic development, children with ADHD differ from children with normal language in their utterance formulation (that is, in the number of pauses, repetitions, revisions, or other such elements), and children with autism typically have pragmatic impairments [24-26]. Nevertheless, some children with autism exhibit language impairments that are more frequently observed in children with SLI and *vice versa*[22]. Given the genetic and phenotypic overlaps between SLI, autism, and ADHD and the HLA linkage and associations identified in studies of dyslexia, autism, and ADHD, we decided to investigate the possible involvement of HLA loci in SLI. Our initial investigations involved the completion of quantitative and case-control association analyses with single-nucleotide polymorphisms (SNPs) across the HLA region. We went on to impute HLA types from SNP array data and tested for association with language impairment, again within quantitative-trait and case-control association models.

## Methods

### Participants

The SLI cohort in this study included families from five centers across the UK—the Newcomen Centre at Guy’s Hospital (London) (now called the Evelina Children’s Hospital), the Cambridge Language and Speech Project (CLASP), the Child Life and Health Department at the University of Edinburgh, the Department of Child Health at the University of Aberdeen, and the Manchester Language Study—as described in previous SLI Consortium (SLIC) studies [14,17,27-29]. In addition, this study included 49 newly-collected families from the Evelina Children’s Hospital cohort which had not been included in previous SLIC studies. All individuals were self-reported white British. These collections were approved by the relevant local ethics committees, and all subjects provided informed consent for participation in this study. All SLIC families were ascertained on the basis of a single proband with expressive and/or receptive language skills at least 1.5 standard deviations (SDs) below that expected for their age (population mean = 100, SD = 15) and Wechsler Intelligence Scale for Children (WISC) Perceptual Organization Index (a composite score of the non-verbal subtests Picture Completion, Picture Arrangement, Block Design, and Object Assembly) of more than 77.5 (1.5 SD below that expected for their age). Probands did not have a diagnosis of autism or hearing impairment. DNA and phenotypic data were collected from all children regardless of linguistic ability, and DNA was collected from all available parents. All probands met the above criteria for SLI, but we did not exclude probands on the basis of a diagnosis of ADHD or dyslexia alone, given the high degree of co-occurrence of SLI and ADHD or dyslexia. However, for some of our SLIC samples, data were available for the presence of hyperactivity, coordination, and reading problems. From this, we estimate that approximately one third of our SLIC samples showed some evidence of ADHD or developmental coordination disorder and that approximately one half of our probands had reading problems.

### SNP arrays and quality control measures

SNP data were generated for both SLIC and control samples by using Illumina Omni-Express (version 12.1) genome-wide SNP arrays (Illumina, San Diego, CA, USA). Prior to HLA imputation, all genotypes were filtered on the basis of several quality-control measures as suggested in [30]: samples with a genotype success rate below 95% or a heterozygosity rate ±2 SD from the mean were removed, as were SNPs with a minor allele frequency of less than 1%, or a Hardy-Weinberg equilibrium *P* value of less than 0.001. Inheritance data within families were used to exclude SNPs and samples with an error rate of greater than 1%. Control data (CEU, YRI, CHB, JPT, Hapmap release #3) were employed through a principal component analysis to exclude individuals with divergent ancestry (although some minor differences due to population substructure may still be present), and samples with known chromosomal rearrangements or discordant sex information (where appropriate) were removed. Genotypes were compared with existing SNP genotype data for 700 individuals across 734 SNPs, yielding an estimated genotype error rate of 0.00967.

### Imputation and grouping of HLA types

HLA alleles for *HLA-A, HLA-B, HLA-C, HLA-DQA1, HLA-DQB1*, and *HLA-DRB1* were imputed from SNP data across chromosome 6 with HLA*IMP:01 [31]. HLA*IMP:01 employed 208 SNPs from across chromosome 6 which it deems informative to impute HLA types for the six HLA loci. The HLA*IMP algorithm first selects informative SNPs that were typed in both the reference and the sample sets. The client program performs several quality-control measures on the SNP data. Genotypes are aligned and phased within HLA*IMP by using a reference panel (HapMap3 CEU b36). The HLA types are then imputed on the HLA*IMP server.

HLA*IMP provides a probability value Q (maximum of the posterior probability distribution, which includes all the probabilities assigned to each allele of a given HLA gene) that may be used as a quality measure. We kept imputations with a Q score of 0.9 or above. The resolution of the HLA*IMP imputations is a four-digit HLA type. To reduce the number of alleles in the analyses while still retaining antigen-specificity distinctions, we grouped the HLA types according to the HLA Dictionary, based on their serological equivalence [32], as previously employed in similar studies [33]. For simplicity, the serological groups will hereafter be referred to as alleles. *HLA-DQA1* types were not grouped, since *HLA-DQA1* types were not included in the HLA Dictionary, and they are presented with their four-digit designations (for example, HLA-DQA1*0501). Alleles that otherwise did not have a WHO assignment were not used in this study.

A comparison of the imputations for a subset of the People of the British Isles (POBI) individuals who were typed for several HLA loci through direct genotyping (samples included in [34]) showed a concordance rate of 91.2% between the genotypes and imputations with Q ≥ 0.9 (122 alleles across all six loci were used in this comparison). It should be noted that the samples were genotyped and not serotyped, and a two-digit resolution for the HLA types was used.

### Quantitative analyses

Three linguistic test scores were investigated by quantitative association analyses. Age-normalized composite scores of expressive and receptive language ability were obtained from the revised version of the Clinical Evaluation of Language Fundamentals (CELF-R) [35]. These scores provide a broad overview of two main components of linguistic ability and have previously been shown to represent consistent phenotypes for quantitative linkage and association analyses of SLI [14,16]. A 28-item NWR test [36] was also administered. NWR may be used as a measure of phonological short-term memory (the ability to retain verbal or speech-based information for a short period of time), and it has also been proposed to be an endophenotype of SLI [37], although its sensitivity is disputed by some [38]. These measures were collected for all SLIC children regardless of language ability. Table 1 contains the descriptive statistics for the language traits of the sample used in this study, and the distribution plots can be found in Additional file 1. For an extensive description of the cohort statistics, see [14,17,27,29]. Age-normalized scores were used for all samples, regardless of language ability. Nine hundred fourteen individuals from 280 language-impaired families were included in the quantitative analyses of both SNP data and HLA types. SNP association analyses were performed using a likelihood-ratio test (“assoc” function) in MERLIN [39]. The analyses included 6,725 SNPs encompassing the HLA region on chromosome 6 (25-35 MB, hg18). Since the imputed HLA types were multi-allelic, quantitative transmission disequilibrium test (QTDT) [40] was used for the family-based quantitative association analyses of these data. The orthogonal “within family” model was used, and the tests considered environmental, polygenic, and additive major gene effect variance components. Three tests were run: a general QTDT and two tests of parent-of-origin effects (maternal and paternal). We also tested for significant differences between the maternal and paternal associations in QTDT.

**Table 1:** Descriptive statistics for the language phenotypes of the children in our sample

**Trait**	**NWR**	**ELS**	**RLS**
Mean	90.858	77.812	88.005
Standard deviation	19.406	16.742	17.354
Median	94	76	87
Minimum	55	45	50
Maximum	136	131	138

ELS, expressive language score; NWR, nonword repetition; RLS, receptive language score.

### Case-control analyses

In addition to quantitative tests of association, we performed case-control analyses to compare HLA allele frequencies between independent probands and population controls. These analyses included 241 independent probands and 566 population controls. To control for non-independence, case-control analyses used only the proband from each family, even if the sibling met criteria for SLI (as described in the Participants section). The population controls came from the POBI study [34] and an Oxfordshire study of gene expression in primary immune cells [41] and were included in the principal component analysis mentioned earlier. All control samples were from adults who were unselected in terms of their language performance, and we had no phenotype data for them. SNP-based case-control analyses were performed using the standard case-control analysis based on a chi-squared test (“assoc” function) in PLINK [42]. SNP-based analyses included 6,725 SNPs encompassing the HLA region on chromosome 6 (25-35 MB, hg18). Since the imputed HLA-types were multi-allelic, we used a Fisher’s exact test calculator [43] to generate *P* values for differences in allele counts between cases and controls.

### Calculation of relative risks for HLA data

Relative risks were calculated for HLA alleles that were significantly associated with increased or decreased risk in the case-control analysis. Samples with one or more unknown alleles (at a given locus) were excluded from these analyses. The calculations of relative risks and confidence intervals were performed by using the method described in [44].

### Multiple testing of SNP and HLA data

In the quantitative analyses of HLA types, empirical *P* values were obtained for each of the tests performed with a permutation procedure implemented in QTDT using 1,000 permutations. QTDT performs Monte-Carlo permutations providing a *P* value with a built-in adjustment for multiple testing which is specific to the dataset in hand. Associations with empirical *P* values below 0.05 are marked by a superscript ‘a’ in Table 2. This method was chosen, as QTDT may test different numbers of alleles in each analysis, depending on the availability of informative families, which is taken into account through the built-in adjustments. In the case-control analysis of HLA-types as well as all the analyses which used the SNP data, the false discovery rate (FDR) method [45] was used to control for the proportion of false positives among the significant results. QVALUE, which uses the model proposed by Storey [46], was used for generating FDR q values for the alleles of each HLA locus separately (with λ = 0). The q values for SNP associations and HLA allele associations with *P* values below 0.05 are reported in the Results section below. This approach is particularly suitable for analyzing a large number of alleles, as using a Bonferroni correction in such cases would be highly over-conservative, resulting in a great reduction in power [47].

**Table 2:** Results of the quantitative transmission disequilibrium test analyses

**HLA allele**	**General QTDT**	**Maternal parent-of-origin analysis**	**Paternal parent-of-origin analysis**
HLA-A A1	0.004 (NWR)+^a^ 0.026 (ELS)+		
HLA-A A3	0.006 (ELS)−^a^		
HLA-B B8		0.021 (RLS)−^a^	0.013 (RLS)+^a^
HLA-C Cw6			0.019 (ELS)−^a^
HLA-C Cw8	0.039 (ELS)+^a^		
DQA1*0301			0.03 (ELS)− 0.011 (RLS)−^a^
DQA1*0501		0.034 (RLS)−^a^	0.029 (RLS)+
DRB1 DR7	0.019 (RLS)+		
DRB1 DR17		0.028 (RLS)−	

Associations with *P* ≤0.05 are shown. The + and − symbols indicate the direction of the association, and the associated trait is listed next to the *P* value. ^a^Associations with empirical *P* ≤0.05 in the permutation analyses. ELS, expressive language score; NWR, nonword repetition; QTDT, quantitative transmission disequilibrium test; RLS, receptive language score.

## Results

### Quantitative association analysis with SNP data

The most associated SNPs in the analyses of the ELS and RLS traits (rs1319972, *P* = 7.72 × 10^−5^ and rs1233579, *P* = 0.00026, respectively) did not fall within coding genes. The most associated SNP in the NWR analysis (rs17266491) fell within the gene *LRRC16A* (leucine rich repeat containing 16A) (*P* = 6.86 × 10^−5^) and represented the most significant result of the SNP-based quantitative analyses. Across the six HLA genes (*HLA-A, HLA-B, HLA-C, HLA-DQA1, HLA-DQB1, and HLA-DRB1*), the most associated SNP was rs3823342 in *HLA-A*. This was found with ELS (*P* = 0.01173) and had a q value of 0.205. Investigation of multiple testing using the FDR method yielded minimum q values of 0.057, 0.064, and 0.155 for the ELS, RLS, and NWR traits, respectively. These data indicate that between 5.7% and 15.5% of the highest-ranking SNP-based associations, of which there were 40 for ELS, 46 for RLS, and 6 for NWR, are likely to represent false positives. Regional association plots for the quantitative analyses can be found in Additional file 1.

### Case-control association analysis with SNP data

The SNP with the strongest evidence for association (rs9467476, with *P* = 0.00027) fell within the gene *LRRC16A*. There were five other SNPs within that gene with *P* ≤0.01, but no other association trends were found. This association did not survive multiple testing: the lowest q value was 0.671, indicating a particularly high false-positive rate in these analyses. Across the six HLA genes examined, the most highly associated SNP was rs1058026 in *HLA-B* (*P* = 0.001885). A regional association plot for the case-control analysis can be found in Additional file 1.

### Quantitative association analyses with HLA data

In total, nine alleles in *HLA-A, HLA-B, HLA-C, HLA-DQA1*, and *HLA-DRB1* showed marginal association (nominal *P* ≤0.05). Furthermore, 8 of these 13 associations survived multiple testing, with an empirical *P* ≤0.05, as assessed by a permutation procedure. The most significant association was seen with allele A1 of the *HLA-A* gene, which was associated with increased performance on the NWR test (nominal *P* = 0.004, empirical *P* = 0.017). In contrast, the A3 allele of *HLA-A* was associated with lower ELSs (nominal *P* = 0.006, empirical *P* = 0.016). A marginal association was also found between the Cw8 allele of *HLA-C,* and higher ELSs (nominal *P* = 0.039, empirical *P* = 0.048).

The parent-of-origin analyses highlighted effects of particular interest for the B8 allele of the *HLA-B* gene (minimum nominal *P* = 0.013, empirical *P* = 0.029) and HLA-DQA1*0501 (minimum nominal *P* = 0.029, empirical *P* = 0.057). Both these alleles were associated with lower RLS when inherited from the mother and higher RLS when inherited from the father. Note, however, that the paternal parent-of-origin effects for HLA-DQA1*0501 did not survive the permutation procedure. Borderline associations were also observed for the paternal alleles Cw6 of *HLA-C* with ELS (nominal *P* = 0.019, empirical *P* = 0.037) and HLA-DQA1*0301 with RLS (nominal *P* = 0.011, empirical *P* = 0.035), which were associated with lower language scores. Table 2 includes all quantitative associations with *P* ≤0.05.

### Case-control association analyses with HLA data

**Figure 1:**
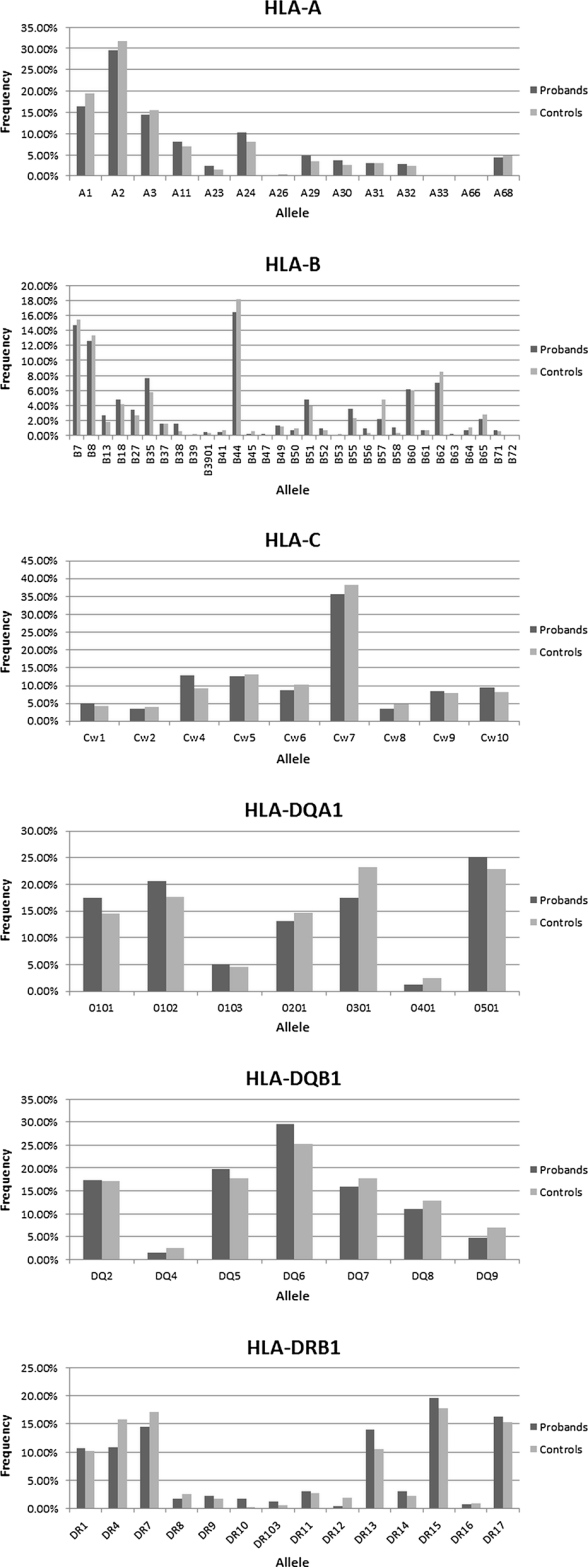
Human leukocyte antigen (HLA) allele frequencies in specific language impairment (SLI) probands and controls.

In total, there were three *HLA-DRB1* alleles (DR4, DR10, and DR12) with nominal *P* ≤0.05. The DR10 allele of *HLA-DRB1* showed a significantly increased allele frequency in probands with language impairment compared with controls (nominal *P* = 0.004), whereas the DR4 and DR12 alleles of *HLA-DRB1* (nominal *P* = 0.02 and nominal *P* = 0.05, respectively) were less frequent in probands with language-impairment than controls indicating a protective risk. FDR analyses indicated that two of these three associations are likely to represent true positives (the q value for the least associated allele was 0.235). The DR10 allele had a particularly strong relative risk of 2.575 (95% confidence interval: 1.773 ≤ relative risk ≤ 3.737), whereas those for DR4 and DR12 were both below 1.0 and the 95% confidence intervals for DR4 did not encompass 1.0. HLA-DQA1*0301 (nominal *P* = 0.014) and the B57 allele of *HLA-B* (nominal *P* = 0.029) were also both significantly less frequent in probands with language-impairment compared with controls. FDR analyses of DQA1*0301 yielded a low q value (0.098), bolstering confidence in its authenticity. In contrast, FDR indicated that the association with *HLA-B* B57 is likely to represent a false positive (q value of 0.911). The relative risk values for DQA1*0301 and B57 were both below 1.0 and had significant 95% confidence intervals that did not encompass 1.0. Table 3 includes case-control associations with *P* ≤0.05 and their relative risks and confidence intervals. Figure 1 includes allele frequencies in probands with language impairment and controls for HLA loci tested.

**Table 3:** Results of the case-control association analysis

**HLA allele**	**Number of alleles counted in probands**	**Number of alleles counted in controls**	**Frequency in probands**	**Frequency in controls**	**Fisher’s exact test P**	**Relative risk**	**95% confidence interval – lower boundary**	**95% confidence interval – upper boundary**	**Effect**
HLA-DRB1 DR10	395	912	0.018	0.002	0.004	2.575	1.773	3.737	Risk
HLA-DQA1*0301	453	1078	0.174	0.231	0.012	0.753	0.59	0.961	Protective
HLA-B B57	443	1055	0.023	0.047	0.029	0.541	0.303	0.963	Protective
HLA-DRB1 DR4	395	912	0.108	0.158	0.02	0.692	0.510	0.939	Protective
HLA-DRB1 DR12*	395	912	0.005	0.02	0.05	0.333	0.09	1.243	Protective

Associations with *P* ≤0.05 are shown. ^a^The *P* value for DR12 was 0.050339 but was included here since the *P* values were rounded up to the third decimal place.

## Discussion

SLI is a complex and heterogeneous neurodevelopmental disorder that affects language development in children. HLA associations have been reported for other neurodevelopmental disorders that show some genetic and phenotypic overlap with SLI. We investigated the possible involvement of HLA loci in SLI using several approaches: quantitative and case-control association analyses both with SNPs in and around the HLA region and with HLA types.

QTDT analyses of HLA-type data identified risk and protective alleles with regard to the three measured traits, and these findings did survive multiple testing. *HLA-A* A1 was the most highly positively correlated allele (with NWR, nominal *P* = 0.004, empirical *P* = 0.017), and *HLA-A* A3 was the most highly negatively correlated allele (with ELS, nominal *P* = 0.006, empirical *P* = 0.016), thus implicating the *HLA-A* locus in susceptibility to SLI.

We also found interesting evidence of parent-of-origin effects. In particular, the *HLA-B* B8 allele and HLA-DQA1*0501 are negatively correlated with RLS when inherited from the mother but positively correlated with the same trait when paternally inherited. Parent-of-origin effects have been reported with regard to the involvement of HLA alleles in neurological disorders such as multiple sclerosis [48], and in this respect it is interesting to find such an effect in SLI. The associations with B8 in both parent-of-origin effects analyses remained significant following the permutation procedure. Interestingly, *HLA-B* has been implicated in schizophrenia; however, the increase in risk was the result of matching *HLA-B* genotypes in the mother and the child [33]. While the association detected in our study is with a specific allele, it is interesting to note that the association is correlated with negative test scores only when the allele is inherited from the mother (so there is a matching of at least one allele between the mother and the child).

Although many of the associations observed in this study were of borderline significance, it is interesting to note that the identified association trends are supported by studies of related neurodevelopmental disorders. In an ADHD study of a Chinese cohort [49], the DR10 allele of *HLA-DRB1* was found to be significantly more frequent in children with ADHD than in controls, as is the case with the probands with SLI in our study. This result translates to a particularly high relative risk of 2.575, with a 95% confidence interval of 1.773 ≤ relative risk ≤ 3.737, and was supported by FDR analyses. Similarly, Wang and colleagues [49] reported that the DR12 allele of *HLA-DRB1* was significantly more frequent in controls than in cases with ADHD, a trend which again is replicated in our study. The trend of association found between the DR4 allele of *HLA-DRB1* in our probands with language-impairment matches that described by Wright and colleagues [50] in a study of schizophrenia but is the opposite of that observed in ADHD by Odell and colleagues [51]. Note, however, that this ADHD association was not replicated in the Chinese study previously mentioned [49]. Interestingly, the *HLA-DRB1* case-control associations observed in the present study also seem to be the opposite to previously described association trends of HLA alleles with autism (albeit not always significantly so). The DR4 allele was associated with autism [52,53] but is significantly less frequent in probands with SLI than in controls (Table 3, Figure 1). DR13 and DR14 (grouped together) have been negatively associated with autism [52], which is, again, the opposite trend to the one observed here (Figure 1, frequency in probands with language-impairment: 0.14 and 0.03; frequency in controls: 0.11 and 0.02, for DR13 and DR14, respectively). Although these differences were not even nominally significant in SLI, they may suggest that if HLA alleles play a role in both SLI and autism, the mechanism itself may be different.

Although quantitative and case-control SNP-based analyses consistently identified associations within the *LRRC16A* gene, none of the SNP-based analyses found strong association trends within HLA loci. Furthermore, the FDR q values obtained for SNP-based associations were all greater than 0.05, indicating a high false-positive rate. Nonetheless, it should be noted that one might not expect to find a direct correlation between SNPs in HLA genes and effects mediated by HLA types, because of the high degree of variation in the HLA region.

## Conclusions

The results of this study suggest a potential involvement of HLA loci in SLI. Quantitative association analyses highlighted *HLA-A* and parent-of-origin effects for *HLA-B*, whereas case-control analyses implicated *HLA-DRB1* alleles. Further, larger-scale studies will be required to replicate these findings. The relatively small sample sizes employed are reflected by nominal *P* values. Since our analyses used imputed HLA alleles and since no HLA associations have been previously reported for SLI, we did not confine ourselves to testing only a subset of the HLA alleles, and, consequently, we performed a relatively large number of tests. Nonetheless, we believe that association testing and imputation of HLA types provide a cost-effective way of studying the involvement of immune-related genes in neurodevelopmental disorders. The preliminary data presented here provide an intriguing link to those described by previous studies of other neurodevelopmental disorders suggesting a possible role for HLA loci in language disorders.

## Abbreviations

ADHD: Attention deficit hyperactivity disorder; ELS: Expressive language score; FDR: False discovery rate; HLA: Human leukocyte antigen; NWR: Nonword repetition (score); POBI: People of the British Isles; RLS: Receptive language score; SD: Standard deviation; SLI: Specific language impairment; SNP: Single-nucleotide polymorphism.

## Competing interests

The authors declare that they have no competing interests.

## Authors’ contributions

RN conceived and designed this study, performed the imputation and association analyses, and drafted the manuscript. NHS helped with the quality control for the DNA and generation of the SNP genotype data. GB managed the acquisition of data and DNA from the Guy’s Hospital SLIC cohort. AO managed the acquisition of data and DNA from the Edinburgh SLIC cohort. GC-R managed the acquisition of data and DNA from the Manchester SLIC cohort. PFB managed the acquisition of data and DNA from the Cambridge SLIC cohort. ERH managed the acquisition of data and DNA from the Aberdeen SLIC cohort; the SLIC is a group of individuals who collected the DNA and data for the SLIC cohort and provided vital intellectual input to the study design and management of the SLIC resource. APM is the principal investigator for the SLIC genetic data and assisted in the conceptualization of the study and contributed to the intellectual content of the manuscript. JCK managed the acquisition of data and DNA from the control individuals from the Oxfordshire study of gene expression in primary immune cells and assisted with the interpretation of the data for the manuscript. BW managed the acquisition of data and DNA from the control individuals for the POBI cohort and provided intellectual input into the study design. SEF managed the generation of SNP data for the SLIC individuals and contributed to the intellectual conception of this study. DFN performed quality-control procedures on the SLIC genetic data and helped with the conceptualization and design of the study and the drafting of the manuscript. All authors read and approved the final manuscript.

## Supplementary Material

Additional file 1 Regional association plots for SNP association and distributions of quantitative traits. Click here for file
