# Supplementary material for: Associations of HLA alleles with specific language impairment
Source: J Neurodev Disord. 2014 Jan 17;6(1):1. doi: 10.1186/1866-1955-6-1 (PMC3906746; doi:10.1186/1866-1955-6-1)

Figures for the association analyses which used SNP data: ELS, RLS, NWR, and case-control. The regional association plots were generated with LocusZoom (Pruim RJ, Welch RP, Sanna S, Teslovich TM, Chines PS, Gliedt TP, Boehnke M, Abecasis GR, Willer CJ. (2010) LocusZoom: Regional visualization of genome-wide association scan results. Bioinformatics 2010, **26**: 2336-2337)

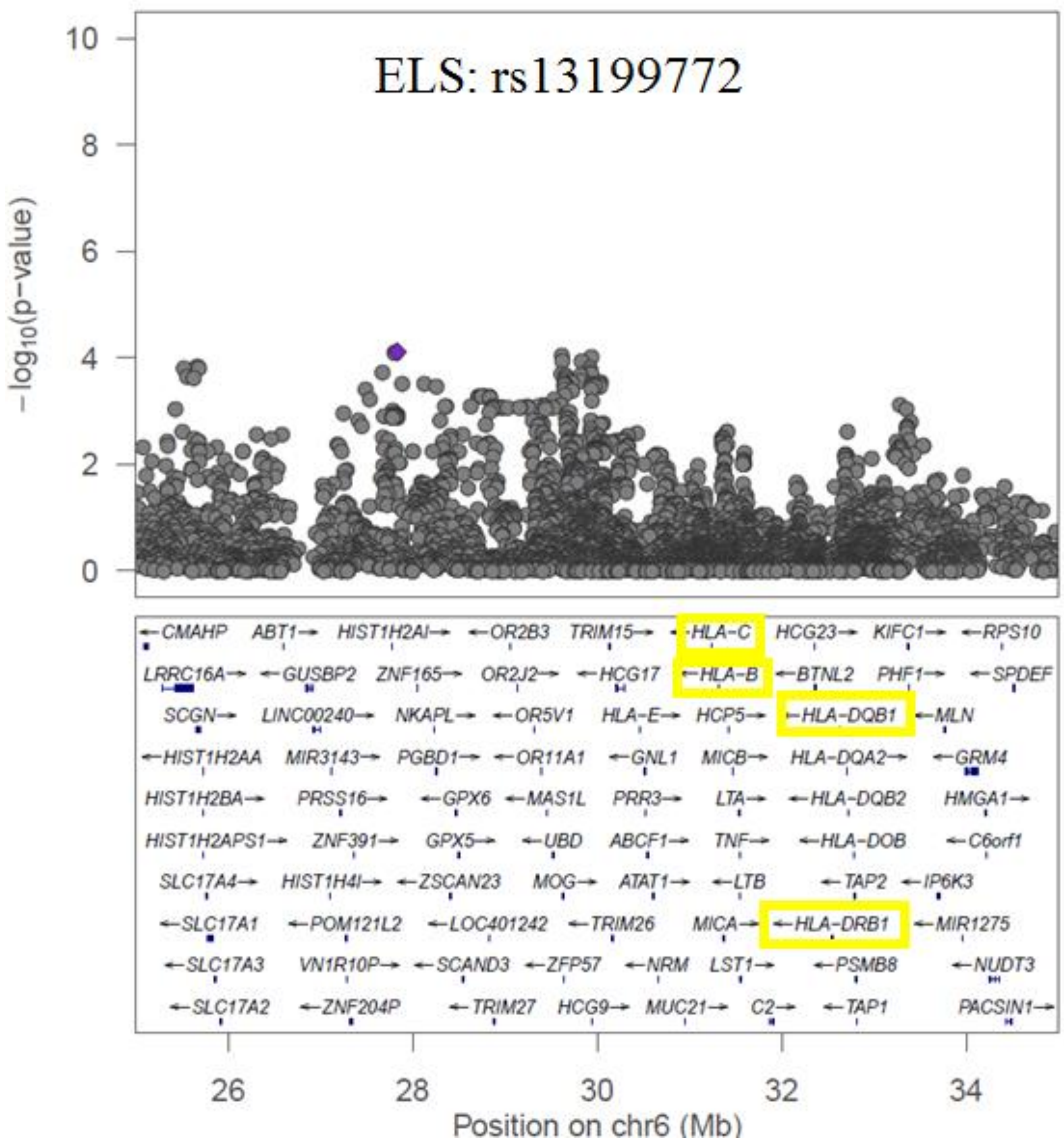

ELS: rs13199772
−log10(p−value)
Position on chr6 (Mb)
CMAHP ABT1 HIST1H2AI OR2B3 TRIM15 HLA-C HCG23 KIFC1 RPS10
LRRC16A GUSBP2 ZNF165 OR2J2 HCG17 HLA-B BTNL2 PHF1 SPDEF
SCGN LINC00240 NKAPL OR5V1 HLA-E HCP5 HLA-DQB1 MLN
HIST1H2AA MIR3143 PGBD1 OR11A1 GNL1 MICB HLA-DQA2 GRM4
HIST1H2BA PRSS16 GPX6 MAS1L PRR3 LTA HLA-DQB2 HMGA1
HIST1H2APS1 ZNF391 GPX5 UBD ABCF1 TNF HLA-DOB C6orf1
SLC17A4 HIST1H4I ZSCAN23 MOG ATAT1 LTB TAP2 IP6K3
SLC17A1 POM121L2 LOC401242 TRIM26 MICA HLA-DRB1 MIR1275
SLC17A3 VN1R10P SCAND3 ZFP57 NRM LST1 PSMB8 NUDT3
SLC17A2 ZNF204P TRIM27 HCG9 MUC21 C2 TAP1 PACSIN1

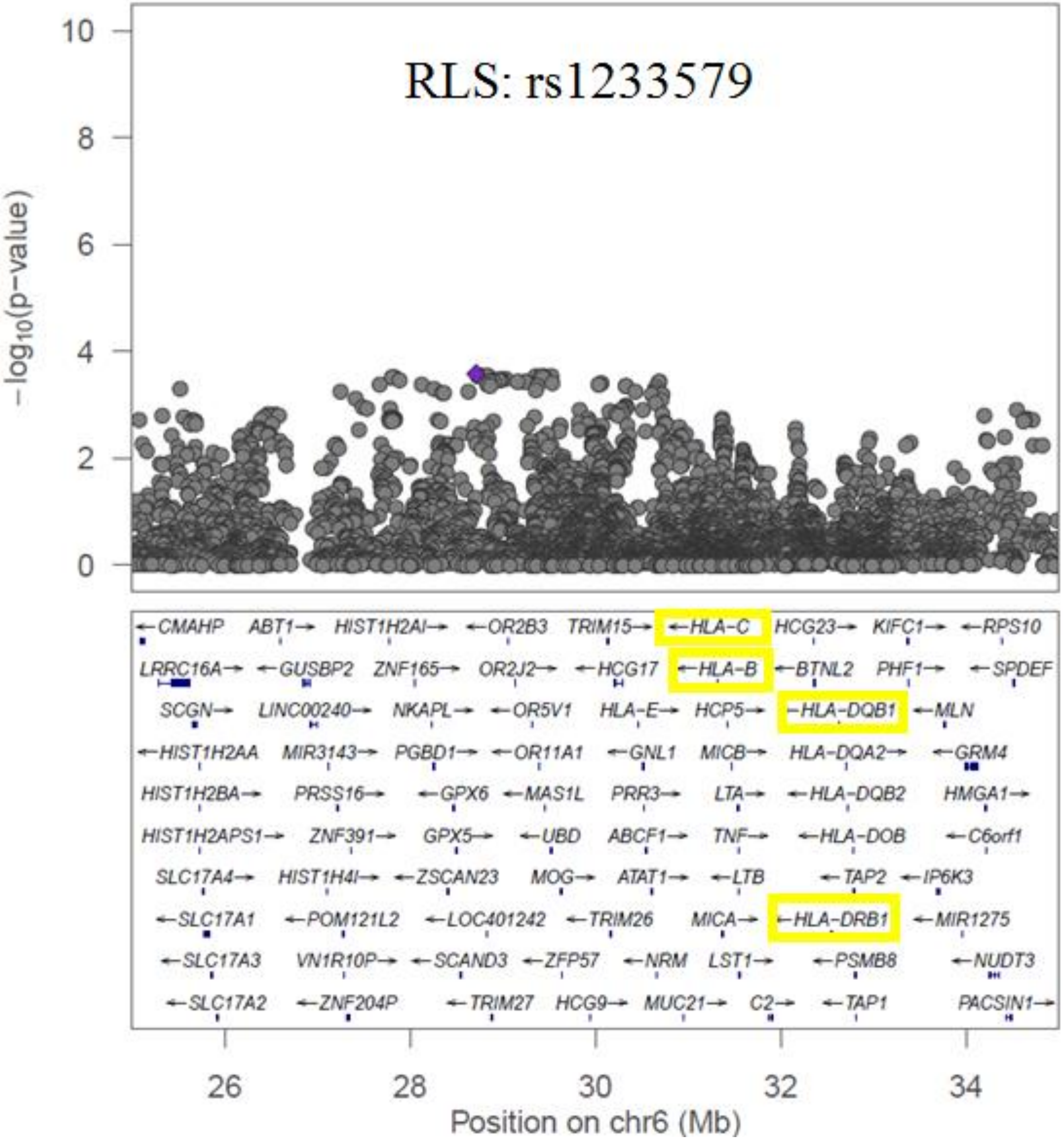
RLS: rs1233579
−log10(p−value)
10
8
6
4
2
0
←CMAHP ABT1→ HIST1H2AI→ ←OR2B3 TRIM15→ ←HLA-C HCG23→ KIFC1→ ←RPS10
LRRC16A→ ←GUSBP2 ZNF165→ OR2J2→ ←HCG17 ←HLA-B ←BTNL2 PHF1→ ←SPDEF
SCGN→ LINC00240→ NKAPL→ ←OR5V1 HLA-E→ HCP5→ ←HLA-DQB1 ←MLN
←HIST1H2AA MIR3143→ PGBD1→ ←OR11A1 ←GNL1 MICB→ HLA-DQA2→ ←GRM4
HIST1H2BA→ PRSS16→ ←GPX6 ←MAS1L PRR3→ LTA→ ←HLA-DQB2 HMGA1→
HIST1H2APS1→ ZNF391→ GPX5→ ←UBD ABCF1→ TNF→ ←HLA-DOB ←C6orf1
SLC17A4→ HIST1H4I→ ←ZSCAN23 MOG→ ATAT1→ ←LTB ←TAP2 ←IP6K3
←SLC17A1 ←POM121L2 ←LOC401242 ←TRIM26 MICA→ ←HLA-DRB1 ←MIR1275
←SLC17A3 VN1R10P→ ←SCAND3 ←ZFP57 ←NRM LST1→ ←PSMB8 ←NUDT3
←SLC17A2 ←ZNF204P ←TRIM27 HCG9→ MUC21→ C2→ ←TAP1 PACSIN1→
26
28
30
32
34
Position on chr6 (Mb)

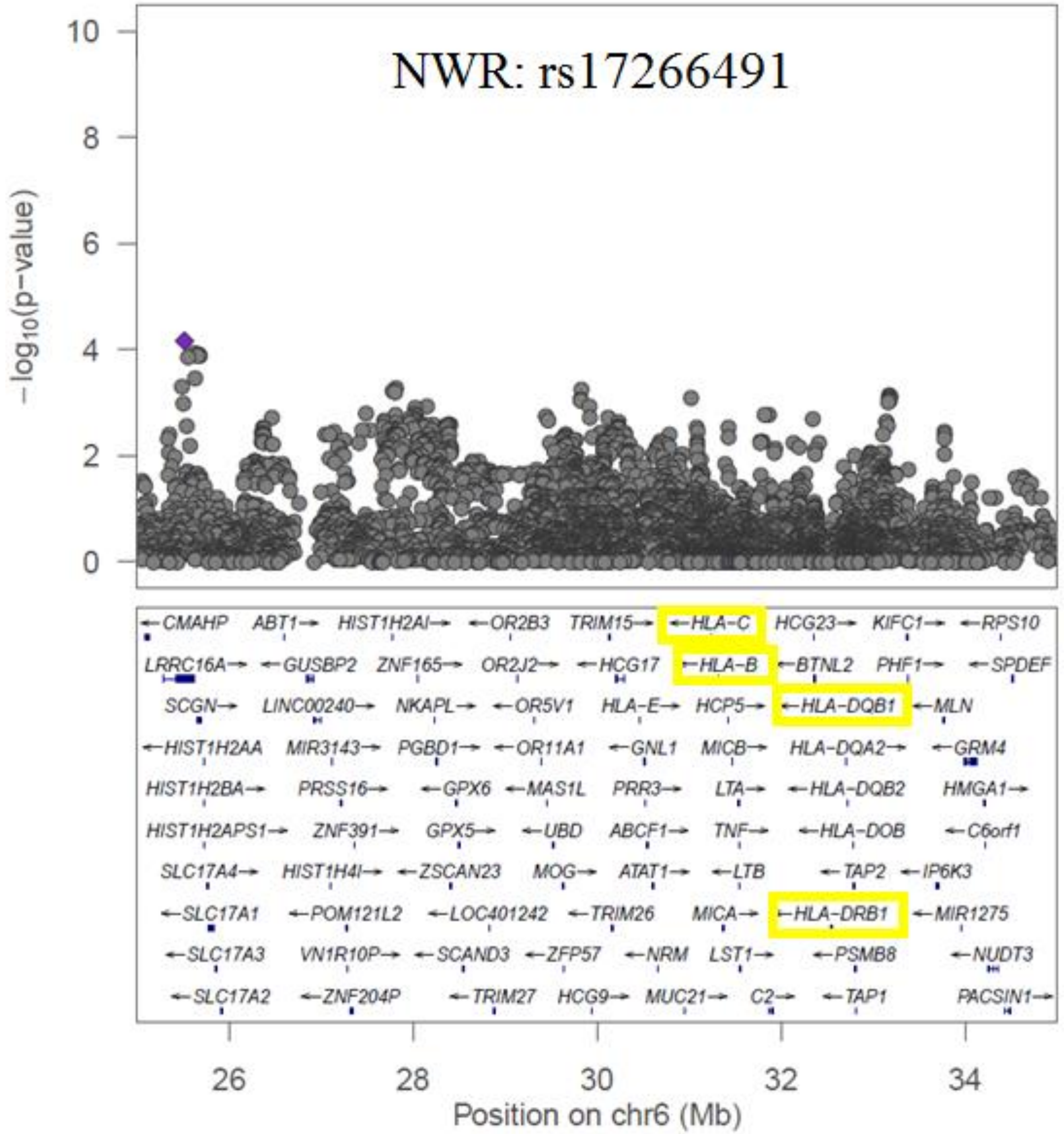

NWR: rs17266491
−log10(p−value)
Position on chr6 (Mb)
CMAHP
ABT1
HIST1H2AI
OR2B3
TRIM15
HLA-C
HCG23
KIFC1
RPS10
LRRC16A
GUSBP2
ZNF165
OR2J2
HCG17
HLA-B
BTNL2
PHF1
SPDEF
SCGN
LINC00240
NKAPL
OR5V1
HLA-E
HCP5
HLA-DQB1
MLN
HIST1H2AA
MIR3143
PGBD1
OR11A1
GNL1
MICB
HLA-DQA2
GRM4
HIST1H2BA
PRSS16
GPX6
MAS1L
PRR3
LTA
HLA-DQB2
HMGA1
HIST1H2APS1
ZNF391
GPX5
UBD
ABCF1
TNF
HLA-DOB
C6orf1
SLC17A4
HIST1H4I
ZSCAN23
MOG
ATAT1
LTB
TAP2
IP6K3
SLC17A1
POM121L2
LOC401242
TRIM26
MICA
HLA-DRB1
MIR1275
SLC17A3
VN1R10P
SCAND3
ZFP57
NRM
LST1
PSMB8
NUDT3
SLC17A2
ZNF204P
TRIM27
HCG9
MUC21
C2
TAP1
PACSIN1

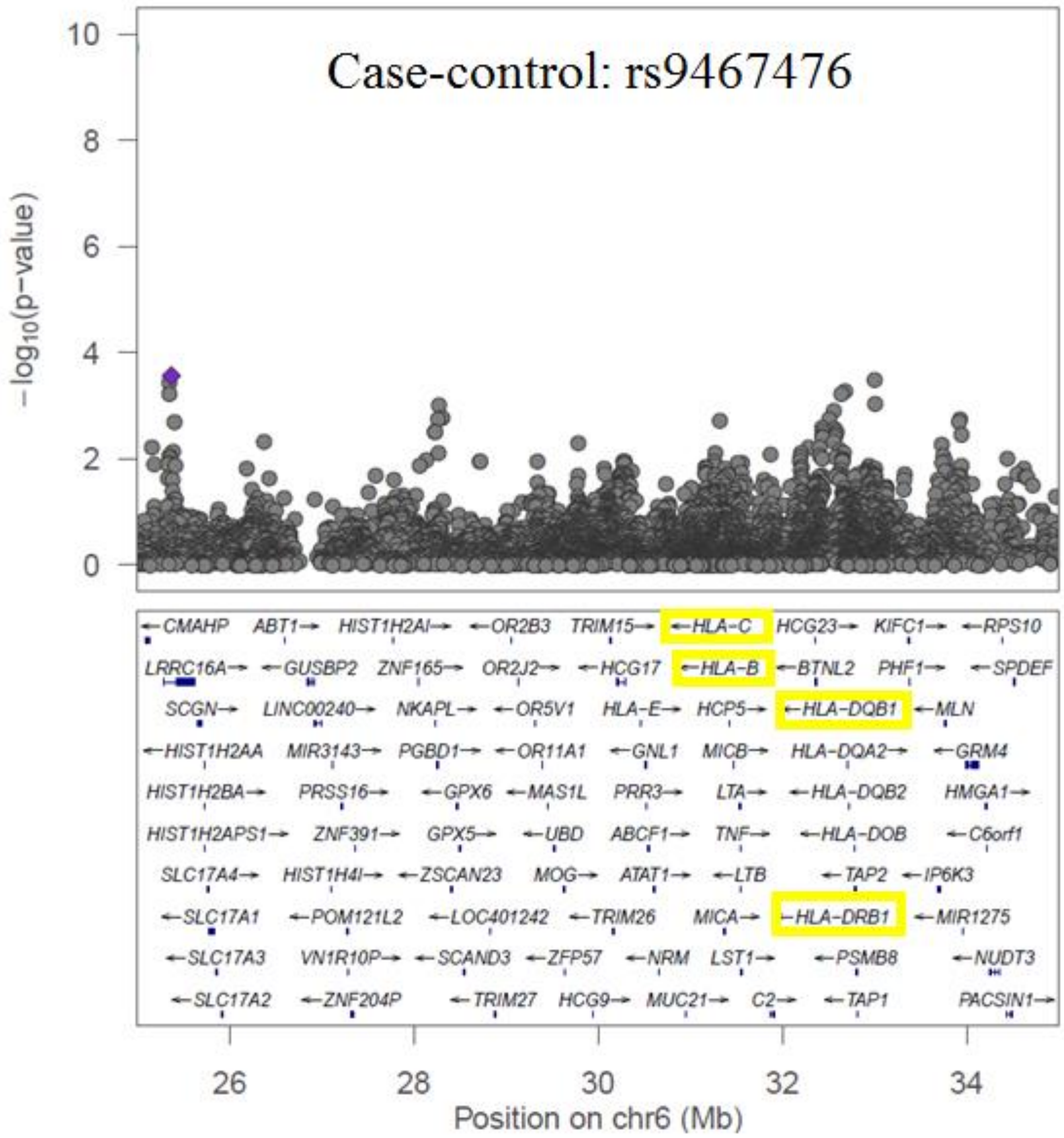

Case-control: rs9467476
−log10(p−value)
10
8
6
4
2
0
CMAHP ABT1 HIST1H2AI OR2B3 TRIM15 HLA-C HCG23 KIFC1 RPS10
LRRC16A GUSBP2 ZNF165 OR2J2 HCG17 HLA-B BTNL2 PHF1 SPDEF
SCGN LINC00240 NKAPL OR5V1 HLA-E HCP5 HLA-DQB1 MLN
HIST1H2AA MIR3143 PGBD1 OR11A1 GNL1 MICB HLA-DQA2 GRM4
HIST1H2BA PRSS16 GPX6 MAS1L PRR3 LTA HLA-DQB2 HMGA1
HIST1H2APS1 ZNF391 GPX5 UBD ABCF1 TNF HLA-DOB C6orf1
SLC17A4 HIST1H4I ZSCAN23 MOG ATAT1 LTB TAP2 IP6K3
SLC17A1 POM121L2 LOC401242 TRIM26 MICA HLA-DRB1 MIR1275
SLC17A3 VN1R10P SCAND3 ZFP57 NRM LST1 PSMB8 NUDT3
SLC17A2 ZNF204P TRIM27 HCG9 MUC21 C2 TAP1 PACSIN1
26
28
30
32
34
Position on chr6 (Mb)

Distributions of quantitative traits in our sample

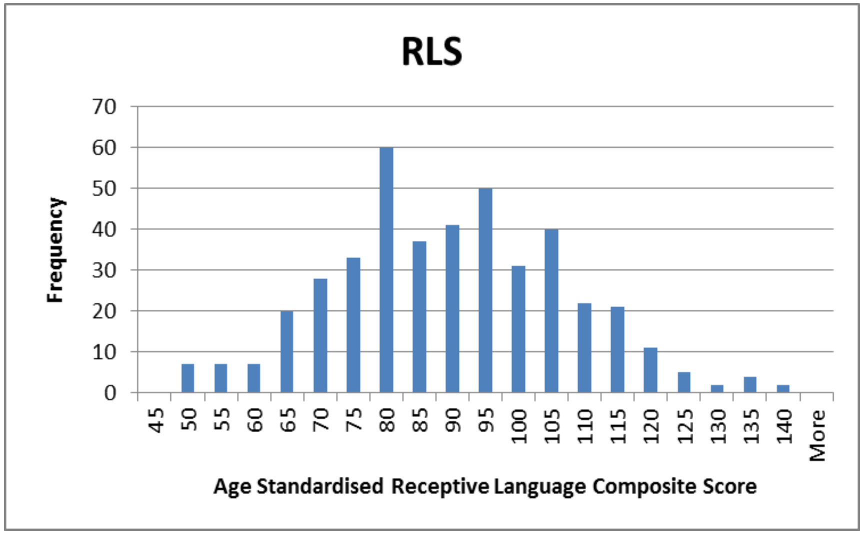

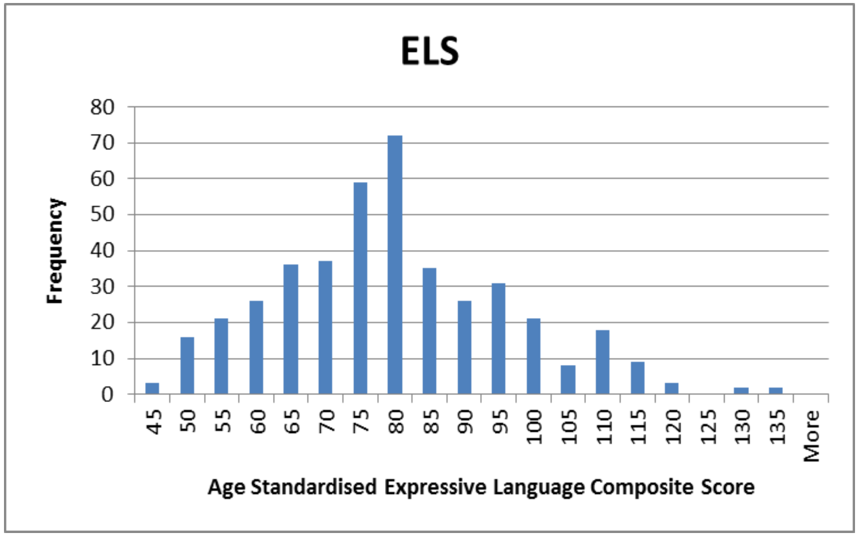

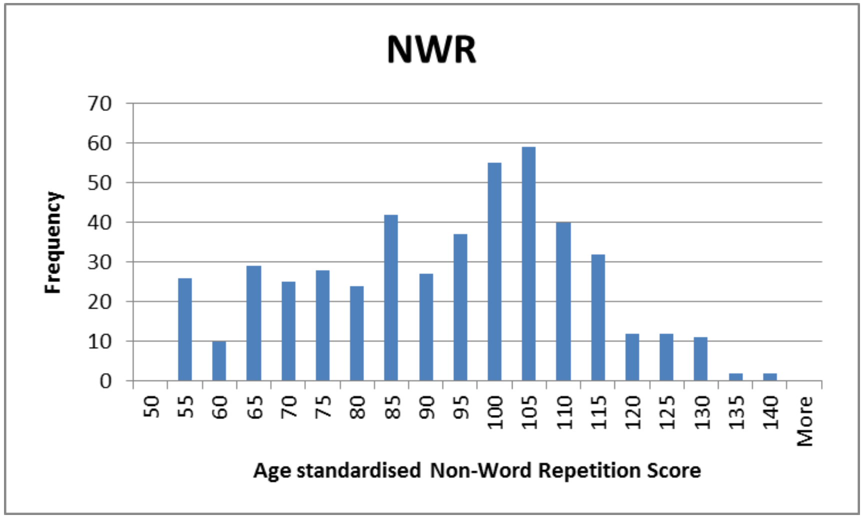

Supplement: Additional file 1 — Regional association plots for SNP association and distributions of quantitative traits. [file 1866-1955-6-1-S1.pdf]
